# The Eldest Female Case of Myasthenia Gravis with an Unusual Presentation: Aspiration Pneumonia as the Initial Manifestation

**DOI:** 10.7759/cureus.19268

**Published:** 2021-11-05

**Authors:** Yuya Masuda, Teru Kumagi, Noriyuki Miyaue, Yuko Hosokawa, Hayato Yabe

**Affiliations:** 1 Postgraduate Medical Education Center, Ehime University Hospital, Toon, JPN; 2 Postgraduate Medical Education Center, Saiseikai Matsuyama Hospital, Matsuyama, JPN; 3 Neurology, Saiseikai Matsuyama Hospital, Matsuyama, JPN

**Keywords:** super-elderly, unusual presentation, myasthenia gravis, etiology, aspiration pneumonia

## Abstract

We report the eldest female case of myasthenia gravis (MG) that initially presented with aspiration pneumonia. A 91-year-old female with a high-grade fever and general malaise who had suffered from expectoration for several years was diagnosed with aspiration pneumonia. Thorough medical history taking and physical examination suggested the possibility of MG as a cause of aspiration pneumonia. Positive acetylcholine receptor antibody and waning phenomenon on a nerve conduction study confirmed the diagnosis. Treatment with intravenous immunoglobulin, prednisolone, and pyridostigmine resulted in a rapid improvement. Physicians should always consider the etiology of aspiration pneumonia to prevent further negative events.

## Introduction

Aspiration pneumonia is a common disease caused by the accidental aspiration of bacteria into the airway along with saliva, food, or gastric juices. It often occurs in elderly people with reduced swallowing function, neurological diseases such as post-stroke syndrome, and Parkinson's disease and in bedridden patients [1]. It accounts for approximately 10% of community-acquired pneumonia and is likely more common in hospital-acquired pneumonia [2]. However, its clinical picture is unclear due to the lack of distinct diagnostic criteria and the heterogeneity of the patient population [1].

Myasthenia gravis (MG) is one of the oldest and best understood autoimmune neurological diseases in medical history [3]. It affects any race with an estimated incidence of 0.3-2.8 per 100,000 population worldwide [4], whereas its estimated incidence in Japan is 23.1 per 100,000 population [5]. Various and combined symptoms, such as dysphagia, dysarthria, limb muscle weakness, and respiratory muscle paralysis, can be observed in patients with MG, but ocular symptoms are typically the first symptoms. However, the onset of dysphagia alone is not common [6].

We herein report a case of aspiration pneumonia in a 91-year-old female eventually diagnosed with MG.

## Case presentation

**Figure 1:**
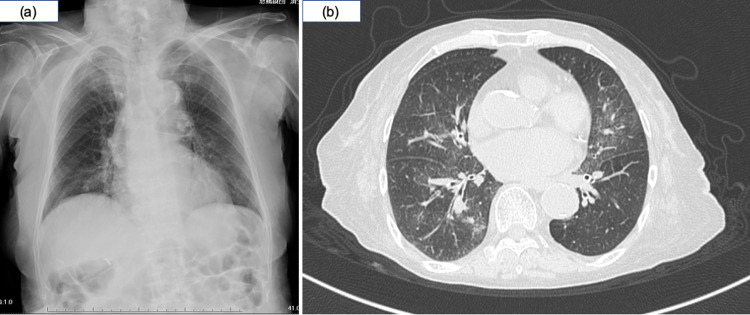
Ground glass opacity in the bilateral lower lobe on chest X-ray (a) and CT (b)

A 91-year-old female was sent to our emergency room due to a high-grade fever and general malaise. She had a history of lacunar infarction without sequelae, hypertension, retinal hemorrhage (left eye prosthesis), and ovarian cyst. She had been expectorating for several years, but the family member simply considered this as her aging habit. However, the amount of her sputum increased from the night before the hospital visit, and she had a fever in the 38°C range from noon on the same day. On arrival, her SpO_2_ was 90%. No abnormalities were found in other vital signs. She did not suffer from dyspnea and cough. On physical examination, coarse crackles were heard in bilateral lower lung fields. No other obvious physical abnormalities were observed. Blood tests showed elevated white blood cells of 14,240/μL (neutrophil: 92.7%) and C-reactive protein of 6.95 mg/dL. Chest X-ray and CT showed a ground glass opacity in the bilateral lower lobe (Figure 1). The patient was diagnosed with pneumonia and was hospitalized. Since chest X-ray and CT also revealed mild mediastinal widening, heart failure was also raised in the differential diagnosis. However, an echocardiogram performed in the emergency room showed preserved cardiac function.

**Figure 2:**
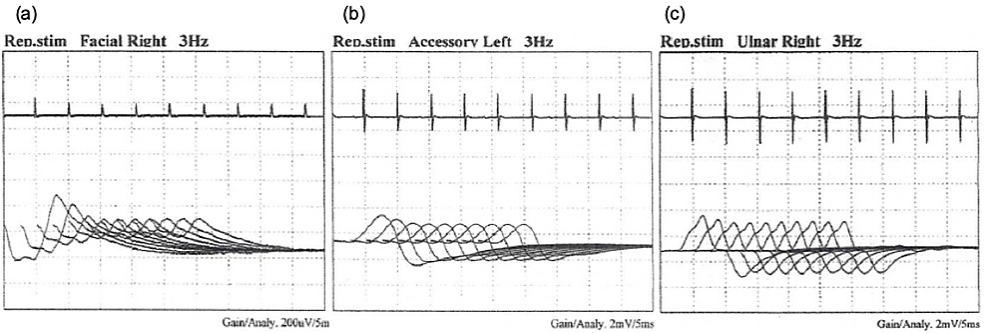
Waning phenomenon with a 3 Hz repetitive stimulation on a nerve conduction study at facial nerve (a), accessory nerve (b), and ulnar nerve (c)

After admission, tazobactam/piperacillin was started, and her fever quickly resolved. Her blood culture was negative, and her sputum culture revealed indigenous bacteria. However, the patient kept expectorating a lot of sputum, which required suction at midnight. She also seemed to aspirate food, so we treated her with fasting and a peripheral drip infusion. On the evening of her fifth hospitalization day, an attending physician (medical resident YM) noticed that her speech had been unclear and her left eye cleft was narrowing compared with the morning ward round. Workup and differential diagnosis included intracranial disease and amyotrophic lateral sclerosis, but there was no abnormality in the head MRI or needle electromyography. A thorough medical interview revealed that she had been having difficulty moving her tongue for nine months prior to the onset. A nerve conduction study performed on the sixth hospitalization day found a waning phenomenon with a 3 Hz repetitive stimulation (Figure 2). Edrophonium chloride test showed an improvement of slurred speech and narrowing of the left eye cleft. A review of chest CT did not show thymoma. Of note, we did not investigate other autoimmune diseases in our patient since there was no clinical feature to suggest such a condition.

After implantation of a nasogastric tube, the patient was treated with intravenous immunoglobulin, prednisolone, and pyridostigmine on the eighth hospitalization day for strongly suggestive MG, which resulted in a rapid improvement. Several days after the initiation of the treatment, the blood test results were positive for acetylcholine receptor antibodies, and a definite diagnosis of MG was made. The patient was discharged from the hospital on the 29th day of hospitalization after the removal of the gastric tube because there was no problem with oral intake.

## Discussion

We experienced a patient with aspiration pneumonia caused by MG. There are two messages in this particular case. One is that we should always consider the etiology of aspiration pneumonia. The other is that, as with any disease, some patients with MG may have atypical features, e.g., initial presentation and age of onset.

The causes of aspiration pneumonia vary greatly by age group. In younger age groups, the most common causes are cerebral palsy, traumatic brain injury, eosinophilic esophagitis, and neck infections. In older age groups, the common causes are head and neck cancer, gastrointestinal disorders such as reflux esophagitis, and postoperative effects. Those with neurodegenerative diseases such as stroke, Parkinson's disease, amyotrophic lateral sclerosis, and dementia diseases such as Alzheimer's disease are also affected. Neurodegenerative diseases are often complicated with dysphagia, especially in Parkinson's disease, with 41%-87% of patients involved [7]. Aspiration pneumonia could be occasionally life-threatening even among youngsters, let alone the elderly. Therefore, it is important to precisely diagnose the cause of aspiration pneumonia to prevent poor outcomes. In this regard, we were able to diagnose our patient with MG in a timely manner.

However, the diagnosis of MG is not straightforward when hallmark symptoms such as ptosis, ophthalmoplegia, and proximal muscle weakness are absent and when patients present with bulbar palsy alone [8]. Furthermore, the definitive and immediate diagnosis of MG is unrealistic because it takes several days to obtain the acetylcholine antibody result. Indeed, there are only a few reports in English literature on aspiration pneumonia as the initial manifestation in MG. A 17-year-old female who had repeated aspiration pneumonia was diagnosed with MG using the Tensilon test [9]. A 25-year-old female patient was diagnosed with bulbar onset MG [10]. There is a case in Japanese literature that reported an 84-year-old female who developed myasthenic crisis due to aspiration pneumonia [11]. These cases suggest the importance of diligent workup with the collaboration of multidisciplinary teams to identify the underlying cause of recurrent pneumonia and to prevent further aspiration in the long run. In our case, treatment was initiated immediately after assessment for possible MG and then reaching a definitive diagnosis of MG. Therefore, evaluations such as swallowing test, bedside ice test, nerve conduction velocity test, and Tensilon test are essential to give an immediate diagnosis of MG. Although there is evidence that MG may be triggered by infections such as SARS-CoV-2 infection [12], this was unlikely in our case since the presumed onset of MG was prior to the presentation of aspiration pneumonia. Regarding the age of diagnosis with MG, only two patients were over 90 years old (95 and 91 years old), and both were male. Our patient is the oldest female patient with MG, followed by 88 and 87 years old [13,14].

## Conclusions

Aspiration pneumonia is a common disease among the elderly and increases with age. However, aspiration pneumonia in the elderly is often regarded as physiological changes, which may lead to a delay in close examination. Indeed, despite the fact that the patient had been experiencing symptoms of dysphagia for some time in our case, it was overlooked as aging. On the other hand, when a young person develops aspiration pneumonia, a thorough investigation of the cause is immediately performed, considering the possibility of neurodegenerative diseases or gastrointestinal diseases as a hidden etiology. Therefore, physicians should always consider the etiology of aspiration pneumonia even in the super-elderly, and this includes atypical presentations of MG.
